# Complete Nucleotide Sequences and Genome Organization of Two Pepper Mild Mottle Virus Isolates from *Capsicum annuum* in South Korea

**DOI:** 10.1128/genomeA.00411-16

**Published:** 2016-05-19

**Authors:** Seung-Kook Choi, Gug-Seoun Choi, Sun-Jung Kwon, Ju-Yeon Yoon

**Affiliations:** Horticultural and Herbal Crop Environment Division, National Institute of Horticultural & Herbal Science, RDA, Wanju, Republic of Korea

## Abstract

The complete genome sequences of pepper mild mottle virus (PMMoV)-P2 and -P3 were determined by the Sanger sequencing method. Although PMMoV-P2 and PMMoV-P3 have different pathogenicity in some pepper cultivars, the complete genome sequences of PMMoV-P2 and -P3 are composed of 6,356 nucleotides (nt). In this study, we report the complete genome sequences and genome organization of PMMoV-P2 and -P3 isolates from pepper species in South Korea.

## GENOME ANNOUNCEMENT

Pepper mild mottle virus (PMMoV), which belongs to the genus *Tobamovirus* in the family *Virgaviridae*, is one of the major viruses in pepper species (*Capsicum* spp.) in the world (1). Tobamovirus strains isolated from pepper plants have been classified into four subgroups, P_0_, P_1_, P_1,2_ and P_1,2,3_, on the basis of their responses with the corresponding resistance genes controlled by L^1^, L^2^, L^3^, and L^4^, which are considered to be alleles at the locus *L* (2). We described a resistance-breaking pathotype of PMMoV previously, designated PMMoV-P3, that overcomes the *L*^3^-mediated resistance in *C. annuum* and compared the pathogenicity of PMMoV-P3 in paprika cultivars with that of PMMoV-P2 (which overcomes the L^2^ resistance) (3, 4). To determine complete nucleotide sequences of PMMoV-P2 and -P3, viral RNAs isolated from purified virus particles using the SDS/Phenol/Proteinase K method (5) were used as templates for reverse transcription-PCR (RT-PCR) with four pairs of PMMoV-universial primers. Synthesized PCR products covering the complete genomes of PMMoV-P2 and -P3 were cloned into the pCR4-TOPO vector for sequencing (Invitrogen, USA).

Although PMMoV-P2 and PMMoV-P3 have different pathogenicities in some pepper cultivars, the complete genome sequences of PMMoV-P2 and -P3 were composed of 6,356 nucleotides (nt). Each viral genome encodes 4 proteins; 126 kDa, 183 kDa, movement protein, and coat protein including 5′ and 3′ untranslated regions with 69 and 198 nt, as described previously. PMMoV-P2 and -P3 shared high sequence identities of nt and amino acids with eight PMMoV strains isolated from geographically different regions. To the best of our knowledge, these are the complete genome sequences and genome organization of PMMoV-P3 isolates from pepper species in South Korea.

### Nucleotide sequence accession numbers.

The complete genome sequences of PMMoV-P2 and -P3 have been deposited in GenBank under the accession numbers LC082099 and LC082100.
